# Routines for reducing the occurrence of emergence agitation during awakening in children, a national survey

**DOI:** 10.1186/2193-1801-3-572

**Published:** 2014-09-30

**Authors:** Pether K Jildenstål, Narinder Rawal, Jan L Hallén, Lars Berggren, Jan G Jakobsson

**Affiliations:** Department of Anesthesiology and Intensive Care, University Hospital, 701 85 Örebro, Sweden; Department of Anesthesiology and Intensive Care, University Hospital, CAMTÖ, Centre for Assessment of Medical Technology in Örebro, Örebro, Sweden; Department of Anesthesiology and Intensive Care, Institution for Clinical Science, Karolinska Institutet, Danderyds Hospital, Stockholm, Sweden

**Keywords:** Emergence agitation, Premedication, Generalanesthesia, Postoperative pain, Postoperative recovery and volatile anesthetics, Volatile anesthetics

## Abstract

Emergence agitation following anesthesia in children is not uncommon. It is, although generally self-limiting, associated with both patient and parents distress. We conducted a national survey around the management of behavioral and neurocognitive disturbances after surgery/anesthesia including a case scenario about a child at risk for emergence reaction. Premedication with clonidine or midazolam would have been used 58 and 37% of responders respectively. A propofol based anesthesia was the most common anesthetic technique, however sevoflurane or desflurane was an option for 45 and 8% of responders. Before awakening 65% would have administered an opioid, 48% a low-dose of propofol and 25% clonidine. Sign or symptoms of behavioral disturbance was not assessed by standardize assessment tools.

A majority of Swedish anesthesia personnel would undertake some preventive action when handling a child at risk for an emergence reaction, the preventive measure differed and it seems as there is an obvious room for further improvements.

## Introduction

Various forms of emergence reactions, emergence agitation and emergency delirium, are commonly seen during awakening after anesthesia in children. Agitation behavior such as restlessness and mental distress may arise from a number of sources including pain, physiological compromise or anxiety. The emergence delirium (ED) involves disturbance in a child's awareness of and attention to their environment with disorientation and perceptual alterations including hypersensitivity to stimuli and hyperactive motor behavior in the immediate post anesthesia period. The degree may vary but restless agitation, crying, anxiety and disorientation may not only cause major parent distress but also lead to self-injury. There are no strict defined guidelines around how to best avoid the occurrence of emergence agitation. A variety of measures have been suggested. Providing anxiolytic premedication, e.g. midazolam or an alpha-2-agonist such as clonidine or dexmetomidine has been show effective reducing but not eliminating the risk (Zhang et al. 2013). Anesthetic technique has also been a matter of debate and there is a clinical perception at least in Sweden in line with the papers recently published by Kanaya et al. that propofol based anesthesia reduce the incidence (Kanaya et al. 2014). It seems obvious that the anesthetic management of children must focus on safety as well as efficacy. We performed survey among Swedish anesthesiologists and nurse anesthetists including a question about how they would take care of a child that experienced emergence reaction during awakening from a previous anesthesia.

## Methods

After approval from the Ethics Committee, Uppsala, Sweden, a questionnaire was sent by e-mail to Swedish anesthesiologists (n = 1326) and nurse anesthetists (n = 1300).

The questionnaire consisted of 3 sections, questions about subjective preferences of the respondents, for example, “what would you like…?”,. Questions were addressing routines and practices and case scenarios. There was one case scenario around the routines for the handling of a child at risk for emergence reaction.

Case study: Postoperative emergence agitation (PEA) (Acute confusion/disorientation upon awakening) Four-year old boy underwent surgery a month ago for arm fracture with osteosynthesis. After surgery, at the PACU, the boy was agitated. The behaviour continued for about 30 minutes. The mother did not recognize her son's angry behaviour. Earlier anesthesia: The child received no premedication, anesthetized with mask inhalation of sevoflurane and maintenance with oxygen/air, and sevoflurane and pain relief with i.v. fentanyl and oral paracetamol. Now the boy is back to remove the osteosynthesis material in the arm.

## Results

The overall response rate to the survey was 38% (n = 417) and 62% (n = 669) for anesthesiologists and nurse anesthetists respectively; in all 1086 responses were collected. The responses to the case scenario is presented in Table 1. Clonidine was the most commonly used premedication in a child that had experienced severe agitation during emergence from a previous anesthesia. A majority of responders would have chosen an anesthetic technique based on propofol and given additional small dose opioid before awakening. A systematic evaluation and assessment with the Pediatric Anesthesia Emergence Delirium (PAED) scale of children showing signs of irritation during awakening was most rarely performed.

**Table 1:** **Case study postoperative emergence agitation**

This questions has only, yes/no/do not know alternatives	Anesthesiologist (%)	Nurse anesthetist (%)	All (%)
1. How do you deal with anesthesia before, during and after this new operation?			
a Premedication with midazolam	26/58/6	37/44/19	37/51/12
	n = 394	n = 600	n = 994
b Premedication with clonidine	59/31/10	37/27/36	48/29/23
	n = 406	n = 610	n = 1004
c Propofol-based anesthesia	70/24/6	71/15/14	71/19/10
	n = 402	n = 610	n = 1012
d Sevoflurane-based anesthesia	43/49/8	47/34/19	45/42/13
	n = 389	n = 600	n = 989
e Desflurane-based anesthesia	11/82/7	5/75/20	8/78/14
	n = 401	n = 500	n = 901
f Administration of low-dose propofol before waking up of patient	45/45/10	52/24/24	48/35/17
	n = 400	n = 569	n = 969
g Administration of clonidine before waking up of patient	33/52/15	17/42/41	25/47/28
	n = 389	n = 437	n = 826
h Administration of opioid before waking up of patient	62/31/7	69/16/15	65/24/11
	n = 398	n = 500	n = 898
2. Do you use any form of diagnostic kits, e.g. Paediatric Anesthesia Emergence Delirium (PAED) scale at PEA in children?	1/81/18	1/60/39	1/75/24
	n = 407	n = 546	n = 953

Questions 1 and 2 response rates are shown as percentage, respondents had 3 choices.

## Discussion

We found that a majority of responders to our Swedish anesthesia personnel survey would undertake some preventive action when handling a child at risk for an emergence reaction. Lerman and Jöhr (Lerman & Jöhr 2009) commented in their review around anesthesia in children that EA is not a new phenomenon, having been first reported about four decades ago (Lerman & Jöhr 2009). Emergence agitation is, although commonly self-limiting within some 10–20 minutes, associated with major patient, parents and personnel distress. Thus efforts in order to reduce its occurrence is of importance. Small dose of propofol, midazolam, opioid, dexmedetomidine, or clonidine have all been shown be beneficial for its prevention and or management. Almenrader et al. made a similar survey as our in Italy and Uk with a response rate of 21% (Almenrader et al. 2014). They found that Italian anaesthetists used midazolam while in propofol opioid combination was commonly used. We provided the option of midazolam or clonidine premedication both shown to reduce the risk (Zhang et al. 2013). There are also increasing data supporting dexmedetomidine premedication as an safe and effective option in children at risk (Sun et al. 2014). We provided the option for administration of a low-dose propofol at end of anesthesia in line with the suggestion by Kim et al. (Kim et al. 2011). When asked for anesthetic technique and a majority would have chosen a propofol based anesthesia and/or propofol at end of anesthesia. The pharmacology of fast-acting volatile agent is highly suspected in the genesis of this complication, however the strength of the evidence supporting this hypothesis can be argued. A recent meta-analysis by Kanaya et al. (Kanaya et al. 2014) demonstrated that EA in children is less likely to occur after propofol anesthesia compared with sevoflurane anesthesia. This seems somewhat opposed by the Cochrane review by Ortiz et al. (Ortiz et al. 2014). This systematic review somewhat surprisingly concluded that there is insufficient evidence to determine whether intravenous anesthesia with propofol for induction and maintenance of anesthesia in pediatric outpatients undergoing surgery reduces the risk of postoperative nausea and vomiting and the risk of behavioral disturbances compared with inhaled anesthesia, that the evidence to support a difference is of poor quality. There is seemingly a need for further studies around preventive measures as alluded in a recent review (Dahmani et al. 2014). Two out of three would have provided an opioid at end of surgery. It seems obvious that the administration of adequate analgesia is not only a feasible but important clinical option (Bortone et al. 2014). We did not give the option to comment on loco/regional anesthesia. Optimization of pain management by regional blocks is without doubt of value in the pediatric patient.

We had an over all response rate of 52%, and we cannot say to what extent the responders had experience in pediatric anesthesia practice. We did provide multiple choice questions and thus alternatives such as the use of dexmedetomedine or loco/regional anesthesia can not be assessed. Still it seems as there is still room for improvements in order to strengthen the perioperative anesthesia protocols for children. It may be that a risk score based approach similar to that for PONV should be implemented adding preventive measures in accordance to risk.

In conclusion, although a majority of Swedish anesthesia personnel would undertake some preventive action when handling a child at risk for an emergence reaction, the preventive measure differed and it seems as there is an obvious room for further improvements.
